# *Ehrlichia canis* and *Rickettsia conorii* Infections in Shelter Dogs: Seropositivity and Implications for Public Health

**DOI:** 10.3390/pathogens13020129

**Published:** 2024-01-29

**Authors:** Paulo Afonso, Ana Patrícia Lopes, Hélder Quintas, Luís Cardoso, Ana Cláudia Coelho

**Affiliations:** 1 CECAV—Animal and Veterinary Research Centre, University of Trás-os-Montes e Alto Douro (UTAD), 5000-801 Vila Real, Portugal; afonso@ipb.pt (P.A.); aplopes@utad.pt (A.P.L.); accoelho@utad.pt (A.C.C.); 2 Associate Laboratory for Animal and Veterinary Sciences (AL4AnimalS), 5000-801 Vila Real, Portugal; 3 Instituto Politécnico de Bragança, Campus de Santa Apolónia, 5300-253 Bragança, Portugal; helder5tas@ipb.pt; 4 Centro de Investigação de Montanha (CIMO), Instituto Politécnico de Bragança, Campus de Santa Apolónia, 5300-253 Bragança, Portugal; 5 Department of Veterinary Sciences, University of Trás-os-Montes e Alto Douro (UTAD), 5000-801 Vila Real, Portugal

**Keywords:** canine tick-borne diseases, *Ehrlichia canis*, Portugal, *Rickettsia conorii*, shelter, zoonosis

## Abstract

A cross-sectional study was conducted to gain insight into the epidemiology of canine ehrlichiosis and rickettsiosis in northern Portugal. Specific IgG antibodies to *Ehrlichia canis* were analysed using a commercial enzyme-linked immunosorbent assay (ELISA), and antibodies to *Rickettsia conorii* were analysed using a commercial indirect immunofluorescence antibody test (IFAT). A total of 113 dogs from two different shelters were sampled, and seroprevalence values of 0.9% (95% confidence (CI): 0.2–4.8%) for *E. canis* and 9.7 (95% CI: 5.5–16.6%) for *R. conorii* were found. Multiple logistic regression investigated risk factors for seropositivity. The odds ratios (ORs) of *R. conorii* seropositivity were higher for female dogs (OR = 6.429; 95% CI: 1.201–34.407). Dogs seropositive for co-infection (*E. canis* + *R. conorii*) were more frequently observed among females (OR = 7.606; CI 95%: 1.478–39.132) and in Shelter 2 (OR = 18.229; 95% CI: 2.190–151.756). These findings show that shelter dogs in northern Portugal are exposed to *E. canis* and *R. conorii*, which can affect both canines and humans. It is imperative to adopt a One Health approach to educate the public about the hazards of canine zoonoses and develop legislation and procedures to control their spread and preserve public health.

## 1. Introduction

In the past few years, there has been an increasing recognition of the importance of canine vector-borne diseases (CVBD) as a growing threat to the health of both humans and animals across the globe. Factors such as climate change, globalisation, rising international mobility and trade, and the rapid growth of human and canine populations have all played a part in causing a shift in the distribution of CVBD [1,2]. The aetiology of CVBD is multifaceted and can encompass a range of disease-causing agents, such as viruses, bacteria, protozoa and helminths. These harmful pathogens are typically transmitted through vectors like ticks, mosquitoes, fleas and lice [1,2]. *Ehrlichia canis*, a member of the order Rickettsiales and an obligate intracellular Gram-negative bacterium, is the primary causative agent of canine monocytotropic ehrlichiosis (CME), which is a severe and sometimes fatal immunosuppressive disease in temperate and tropical regions of Africa, Europe, and the United States of America (USA). It is transmitted globally by the brown dog tick, *Rhipicephalus sanguineus sensu lato* [3,4,5]. In this vector, *E. canis* transmission is feasible transstadially but not transovarially [6]. In Europe, only the species *E. canis* has been identified in dogs [4].

*Ehrlichia canis* causes a wide range of clinical signs in dogs with infection ranging from subclinical to fatal illness [7]. Common clinical manifestations include anorexia, epistaxis, fever, lethargy, weight loss, other haemorrhagic signs, pale mucous membranes and lymph node enlargement [8].

Rickettsioses, caused by Gram-negative obligate intracellular bacteria also in the order Rickettsiales and transmitted by ticks, represent a relevant causative factor within CVBD [9]. *Rickettsia conorii* is an important causative agent of spotted fever group (SFG) illnesses in the Mediterranean, southern Europe, north and sub-Saharan Africa, and the Middle East. However, in the Americas, Australia and the Far East, other *Rickettsia* spp. are more commonly responsible for SFG illnesses. Mediterranean spotted fever (MSF) is a disease that strikes suddenly, and in humans, it typically causes high fever, flu-like symptoms, a black eschar at the site of the tick bite and a maculopapular rash. In serious cases, the disease may cause severe neurological symptoms and affect multiple organs. The mortality is estimated to be around 2.5%. Elderly age, cirrhosis, chronic alcoholism and glucose-6-phosphate dehydrogenase deficiency are traditional risk factors for severe forms of the disease [10,11]. This seasonal human disease predominantly occurs from April to October, reaching its peak from June to August [10]. The first cases of human infection by *R. conorii* in Portugal were described in 1910 with a disease characterised by high fever and skin spots [12]. The primary vector for *R. conorii* is also the brown dog tick, *R. sanguineus s. l*. [13]. This tick species exhibits a global geographic range, a high capacity for pathogen transmission and a remarkable ecological adaptation [2]. However, other species of *Rhipicephalus* and *Ixodes* ticks may also serve as vectors for *R. conorii* [14]. Due to their high tick exposure, dogs serve as sentinels for human infection. Since dogs live near humans and frequently share the same living space, the presence of seropositive dogs can indicate endemic locations and risk factors for illness occurrence in humans [15].

To confirm a rickettsial infection (including *E. canis* and *R. conorii*), it is necessary to either directly detect the presence of the bacteria through molecular methods or perform serological testing to identify the presence of specific antibodies. However, it is important to note that if the test is conducted too early in the course of the bacterial infection before the production of antibodies, it may yield false negative results [16,17,18].

Shelter medicine plays an important role in the health of animals, people and the environment, making it a compelling example of the One Health concept. This integrated approach recognises the interconnectedness of humans, animals, and environmental health, and it emphasises how they influence each other [19]. Shelter medicine contributes to human health by preventing the spread of zoonotic diseases. Providing medical care and vaccination to shelter animals reduces the risk of these diseases spreading to shelter staff and potential adopters [20]. Shelter medicine in Portugal extends its impact beyond the country’s borders through international adoptions. Many shelters and rescue organisations in Portugal facilitate the adoption of animals by individuals and families from other countries [21,22]. This provides homes for animals and promotes global cooperation in animal welfare and health. Shelter animals, especially dogs, are particularly susceptible to tick infestations and the pathogens transmitted by these vectors, including *E. canis* and *R. conorii* [23,24,25]. These dogs serve as a critical reservoir for these vector-borne agents, potentially contributing to their transmission to other animals and even humans. The confined and often overcrowded conditions within shelter environments can facilitate close contact between infected and susceptible animals, increasing the risk of disease spread [24,26]. Furthermore, as dogs are known to share strong bonds with humans and often become adopted into households, the potential for zoonotic transmission becomes a relevant concern. Thus, shelter dogs play a vital role in the epidemiology of these vector-borne diseases, warranting attention to disease prevention and control measures to safeguard the health of both animals and humans.

Conducting seroepidemiological surveys in shelter animals is essential for the animal’s welfare, reducing the risk of disease transmission to humans and maintaining community health. This circumstance embodies the One Health concept, recognising the interdependence between human and animal health. The present study aimed to conduct a serological survey for *E. canis* and *R. conorii* infections in dogs from two animal shelters in northern Portugal, primarily seeking to answer the following questions: What is the seroprevalence of *E. canis* and *R. conorii* in shelter dogs in northern Portugal? And are there significant regional differences in the prevalence of these infections between the shelters in Braga and Bragança? Additionally, the study investigated whether certain demographic factors (such as the sex and age of the dogs) are associated with higher seroprevalence values. By addressing these specific questions, the study aimed to provide critical insights into the regional epidemiology of these infections, contributing to better-informed veterinary practices and public health policies within the context of the One Health approach.

## 2. Materials and Methods

### 2.1. Study Area

This study was conducted in two shelters, one in Braga district (Shelter 1) and the other in Bragança district (Shelter 2), which are located in northern Portugal. Braga is located in the former province of Minho, and Bragança is part of the historical province of Trás-os-Montes e Alto Douro. The geographical area of northern Portugal spans 21,286 km^2^ and has a resident human population of 3,587,074 inhabitants, according to the 2021 census [27]. The selection of the two shelters in the Braga and Bragança districts was strategic. While both are located in northern Portugal, they exhibit distinct climatic and environmental conditions relevant to vector-borne diseases due to their geographical and topographical variations. Braga, with its maritime temperate climate, experiences mild and wet conditions conducive to a consistent presence of vectors like ticks and fleas. On the other hand, Bragança’s continental climate leads to harsher winters and hotter summers, affecting the seasonal dynamics of vector populations. This selection aimed to provide a representative sample of the canine population in different shelters within the region. Seasonal patterns influenced the choice of the sampling period from March to May. This time frame typically marks the onset of warmer weather in Portugal, which correlates with increased activity of ectoparasites such as ticks and fleas. Sampling during this period is thus more likely to reflect the peak risk of exposure to vector-borne diseases, thereby enhancing the relevance and applicability of the study’s findings in understanding and managing canine vector-borne diseases in sheltered dogs.

### 2.2. Animals and Samples

This study was based on a convenience sample of 113 dogs from two shelters. All these dogs were available for adoption. Dogs were sampled from March to May 2022. Based on a physical examination, veterinarians classified the animals as apparently healthy dogs. All dogs were examined for ectoparasite infestation (ticks, fleas and lice). Blood samples were collected from dogs of the two shelters in the scope of regular testing. Information on the sex, age and location of the shelter for each animal was recorded. Animal history was not available, since all dogs were stray animals. The serological analysis included determining the presence of specific antibodies to *E. canis* by an enzyme-linked immunosorbent assay (ELISA) and *R. conorii* by an indirect immunofluorescence antibody test (IFAT).

For *Ehrlichia* diagnosis, serum samples from dogs were diluted at 1:100 in sample buffer and screened for the qualitative detection of circulating IgG antibodies for *E. canis* with the Euroimmun^®^ test (Euroimmun Medizinische Labordiagnostika AG, Lübeck, Germany). The reported sensitivity/specificity of the Euroimmun^®^ test was 92%/100% for *E. canis*, respectively. This ELISA was operated according to the manufacturer’s instructions in the product package insert. Ratios were stratified into three rising categories: samples with a ratio < 0.8 were considered negative, samples between ≥0.8 and <1.1 were considered borderline, and samples with a ratio ≥ 1.1 were considered positive.

The same samples were further tested by IFAT using commercial IFA slides (MegaFLUO^®^ RICKETTSIA conorii, MEGACOR Diagnostik GmbH, Hoerbranz, Austria) for the detection of specific IgG antibodies to *R. conorii* according to the manufacturer’s instructions. Sera were tested at a cut-off dilution of 1:80, which was considered positive.

### 2.3. Data Analysis

An exact binomial test was used to calculate confidence intervals (CI) for the proportions with a 95% confidence level. Chi-square and Fisher’s exact tests compared proportions of positivity related to categorical dependent variables. A probability (*p*) value < 0.05 was regarded as statistically significant. Case definition: a dog testing positive for *E. canis* or *R. conorii* antibodies was considered infected.

Variables showing a significant difference between categories were selected for multiple logistic regression analysis to identify independent risk factors of exposure to *E. canis* or *R. conorii*, calculating odds ratios (ORs) and their 95% CI. Significant potential risk factors at *p* < 0.05 (two-tailed; alpha = 0.05) were then evaluated using stepwise regression to construct a multiple model (Wald test stepwise *p*-value to enter: *p* < 0.05). The multiple logistic model was developed using a stepwise approach. Backward elimination followed by a forward selection for each variable at a time was performed using a likelihood ratio test at each step with 0.05 (two-tailed; alpha = 0.05) as the significance level for removal or entry. The fit of the models was assessed using the Hosmer and Lemeshow goodness-of-fit test [28]. The model was rerun until all remaining variables presented statistically significant values (*p* < 0.05). All statistical analyses were performed using SPSS^®^ 29.0 software for Windows.

## 3. Results

**Figure 1:**
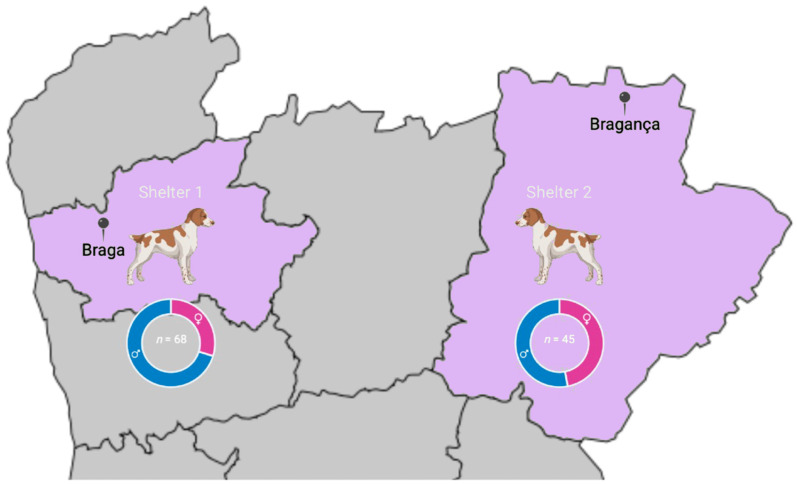
A total of 113 dogs were sampled from two shelters in Braga (Shelter 1) and in Bragança (Shelter 2) districts in northern Portugal.

A total of 113 dogs were studied (Figure 1). There were no detectable ticks in any dogs at visual inspection and no known history of tick exposition despite its possibility. Regarding sex, 45 (39.8%) were females and 68 (60.2%) were males. There were 57 (50.4%) animals aged 12 months or less and 56 (49.6%) older than 12 months. All dogs were mongrels, i.e., they did not belong to any officially recognised breed.

Seroprevalence values of 0.9% (*n* = 1) (95% CI: 0.2–4.8%) for *E. canis* and 9.7 (*n* = 11) (95% CI: 5.5–16.6%) for *R. conorii* were found. Of the dogs tested, 3.5% (*n* = 4) (95% CI: 1.4–8.7%) and 10.6% (*n* = 12) (95% CI: 6.2–17.6%) had inconclusive results for *E. canis* and *R. conorii*, respectively. Twelve dogs were positive for all agents (10.6%; 95% CI: 5.6–17.8%) (Table 1).

### 3.1. Seropositivity to E. canis

Among the *E. canis* positive samples, the prevalence in females (2.2%; 95% CI: 0.06–11.8%) was higher than in males (0.0%; 95% CI: 0.0–5.3%), but the difference was not statistically significant (*p* = 0.173). Regarding age, the prevalence found in dogs with 12 months or less was 0.0% (95% CI: 0.0–6.3%), and in older than 12 months, it was 1.8% (95% CI: 0.04–9.6%), but these differences were not statistically significant (*p* = 0.235). Regarding origin, the lowest value of prevalence was found in Shelter 2 (0.0%; 95% CI: 0.0–7.9%), and the highest value was found in Shelter 1 (1.5%; 95% CI: 0.04–7.9%) with these differences not being statistically significant (*p* = 0.312) (Table 2).

Sex, shelter and age were not found to be significantly associated with seroprevalence of *E. canis*.

### 3.2. Seropositivity to R. conorii

The seroprevalence of antibodies to *R. conorii* was significantly different between females (20.0%; 95% CI: 9.6–34.6%) and males (2.9%; 95% CI: 0.36–10.2%) (*p* = 0.003). When comparing results among different age groups, the prevalence found in dogs with 12 months or less was 5.3% (95% CI: 1.1–14.6%) and 14.3% in dogs older than 12 months (95% CI: 6.4–26.2%), but these differences were not statistically significant (*p* = 0.106). So, age was not associated with the seroprevalence of *R. conorii*. Regarding origin, the lowest value of prevalence was found in Shelter 1 (0.0%; 95% CI: 0.0–5.3%) and the highest was found in Shelter 2 (24.4%; 95% CI: 12.9–39.5%) (Table 2) with these differences being statistically significant (*p* < 0.001).

### 3.3. Seropositivity to Co-Infections (E. canis + R. conorii)

The seroprevalence of antibodies to co-infections (*E. canis* + *R. conorii*) was significantly different between females (22.2%; 95% CI: 11.2–37.1%) and males (2.9%; 95% CI: 0.36–10.2%) (*p* = 0.001). When comparing results among different age groups, the prevalence found in dogs with 12 months or less was 5.3% (95% CI: 1.1–14.6%) and 16.1% in dogs older than 12 months (95% CI: 7.6–28.3%), but these differences were not statistically significant (*p* = 0.057). So, age was not associated with seroprevalence of co-infection (*E. canis* + *R. conorii*). Regarding origin, the lowest value of prevalence was found in Shelter 1 (1.5%; 95% CI: 0.04–7.9%) and the highest was found in Shelter 2 (24.4%; 95% CI: 12.9–39.5%) (Table 2) with these differences being statistically significant (*p* < 0.000).

### 3.4. Risk Factors for R. conorii and the Co-Infection (E. canis + R. conorii) in Sheltered Dogs

Univariable models results in shelter dogs are shown in Table 3. Two variables were associated (*p* < 0.05) with seropositivity to *R. conorii* in shelter dogs in the univariable model. The odds of *R. conorii* seropositivity were found to be higher for female dogs (OR = 2.32; 95% CI: 1.58–3.39) and for dogs belonging to Shelter 2 (OR = 3.0; 95% CI: 2.28–3.95).

Dogs seropositive for co-infection (*E. canis* + *R. conorii*) were more frequently observed among females (OR = 2.4; CI 95%: 1.66–3.47) and in Shelter 2 (OR = 2.72; 95% CI: 1.97–3.76).

For multiple logistic regression analysis, the forward elimination procedure was used to eliminate the factors that were not significant at *p* < 0.05 in the overall model. Those variables with *p* < 0.05 (adjusted OR, 95% CI) were considered as significant potential risk factors for *R. conorii* antibody seropositive results and for co-infection (*E. canis* + *R. conorii*) seropositive results.

The multiple logistic regression analysis of the OR for being seropositive to potential risk factors is presented in Table 4. At the individual level, the odds of *R. conorii* seropositivity were found to be higher for female dogs, i.e., OR = 6.429 (95% CI: 1.201–34.407). The final multiple logistic regression model showed that the odds of co-infection (*E. canis* + *R. conorii*) seropositivity were found to be higher for female dogs, i.e., OR = 7.606 (95% CI: 1.478–39.132) and dogs from Shelter 2, i.e., OR = 18.229 (95% CI: 2.190–151.756).

## 4. Discussion

Our study conducts a comprehensive evaluation of the prevalence of *E. canis* and *R. conorii* infections in shelter dogs in northern Portugal. Unlike previous studies that focused predominantly on owned dogs or on those with clinical signs, our research uniquely targeted a shelter dog population. This approach is relevant because shelter dogs, often overlooked in veterinary research, can serve as crucial sentinels for vector-borne diseases due to their diverse backgrounds and exposure risks. Additionally, our study employed both ELISA and IFAT methods for a more accurate and detailed understanding of the seroprevalence of these infections, contributing to a deeper comprehension of the epidemiological landscape of CVBD in this region. This research fills a gap in the current knowledge and has implications for public health and veterinary practices, emphasising the need for regular screening and prevention strategies in shelter environments.

The present results reveal the existence of antibodies to *E. canis* and *R. conorii* in dogs in the study area. The seroprevalence of *E. canis* in our study was 0.9%. Another study carried out in dogs in 120 veterinary medical centres from all the regions of mainland and insular Portugal, using an ELISA rapid test, reported an apparent seroprevalence of 0.7% in northern Portugal. This same study found an overall seroprevalence of 4.1% in apparently healthy dogs and 16.4% in dogs suspected of CVBD all over the country [29]. In northern Portugal, molecular techniques previously confirmed the infection in dogs with clinical signs [30,31]. Other epidemiological studies performed by molecular techniques confirmed the infection in central [32] and southern Portugal [33,34]. In Portugal, there is also a molecular report of *E. canis* infection in foxes [35]. The individual seroprevalence found in dogs in the present study was similar to previously reported values in northern Portugal, but it was much lower than those found in other studies in the country. Our findings are higher than the lower seroprevalence in some European countries, ranging from 0.2% in Hungary [35] to 0.3% in Finland and France [36,37]. On the other hand, our findings are apparently lower than the reported seroprevalences among dogs in Europe. Previous studies reported seroprevalence values of 0.9–10.1% in Germany [38], 2.1% in Romania [39], 3.1–19.2% in Spain [40], 6.4–46.7% in Italy [41], 11.1% in Serbia [42], 17.9% in Albania [43], 20.7% in Turkey [44], 58.3% in Greece [45] and 29.8% in a shelter and 12.3% in owned dogs in Montenegro [46].

Other studies outside Europe performed with molecular techniques found values ranging from 1.9% to 5.8% in Angola [47,48] to 3.1% in Qatar [49].

In the present study, we found an overall seroprevalence of 9.7% for *R. conorii*, indicating some history of exposure to or active infection with this pathogen. This value is much lower than previous findings in Spain. Prior studies recorded a prevalence of 56.4% in northeastern Spain [50] and 24.6% in the northwestern part of the country [40]. Studies performed in Italy, an endemic country, reported anti-*R. conorii* antibodies in dogs with seroprevalences ranging from 15.5% to 74% [41,51]. A study on police dogs reported a seroprevalence of 72% in Albania [43]. Other seroprevalences in dogs range from 23% in Croatia [52] to 44.8% (in hunting dogs) in Serbia [53]. In the study performed in Montenegro, the prevalence of *R. conorii* was higher in owned dogs (81.9%) than in dogs from a shelter (60.6%) [46].

We additionally studied other risk factors for seropositivity. The ORs of being positive for *R. conorii* and co-infection were significantly higher in females than males. Our results do not agree with previous reports. One specific study has reported higher seropositivity in males due to their increased likelihood of contact with tick species compared to females, owing to behavioural characteristics [54].

The present study found an overall seroprevalence of 10.6% for co-infections. Previous studies indicated that co-infections in vertebrate hosts, often arising from concurrent or sequential exposure to distinct tick species or the transmission of multiple pathogens by a single tick species, are frequent and can complicate both diagnosis and treatment, potentially increasing the likelihood of severe disease. Typically, co-infections involve pathogens that share a common vector and/or have overlapping geographical ranges [55,56,57].

Concerning ehrlichiosis, there is no long-lasting or adequately efficient immune response to ensure the host’s protection in the event of recurring infection [58]. Distinguishing between potential reinfections and persistent subclinical conditions is difficult. In most cases, dogs are returned to the environment where they lived before infection, creating conditions for repeated exposure to infected ticks. This circumstance emphasises the need for planned tick management strategies to keep susceptible dogs safe [59].

Our results showed no difference between dogs’ age and seropositivity to the studied pathogen species. This finding is not in line with previous studies [24,60], which found an association between older dogs and seropositivity, as antibodies to *R. conorii* can remain detectable for a long period [61]. Elderly dogs have a higher likelihood of exposure throughout their lives, and dogs in suboptimal physical conditions may experience a weakened immune system, which increases the risk of infection [24,60].

In dogs with acute disease, PCR techniques for *E. canis* DNA are more sensitive than ELISA or IFAT for the early detection of CME. For the routine diagnosis of *E. canis* infection, PCR tests are commonly accessible. Several laboratories provide panels that include PCR assays for various vector-borne diseases. *Ehrlichia canis* PCR tests can be conducted on blood, lymph node aspirates, splenic aspirates or bone marrow. For the diagnosis of chronic CME, convalescent ELISA or IFAT are far more sensitive than PCR assays [62,63].

The obtained results should be analysed with attention. An ELISA or IFAT-positive result only indicates a past or present infection and does not necessarily reflect the current disease status. A positive result can be obtained based on antibody titres even if the condition has been resolved, as antibodies may persist in the body for several months or even years after initial infection [50]. Regardless of whether an active infection is present, an animal may be serologically negative, especially during the incubation period or the early stages of the illness. In ehrlichiosis, antibody synthesis typically commences 12 to 14 days after infection [50,64,65].

The presence of inconclusive results for *E. canis* (3.5%), *R. conorii* (10.6%) and co-infection (14.2%) in our study warrants special attention, as these outcomes may have considerable implications for the overall conclusions of the research. Firstly, the inconclusive results for both *E. canis* and *R. conorii* suggest the possibility of subclinical exposure or infection in these dogs, which could affect the assessment of the true prevalence of these pathogens in the studied population. This ambiguity in the data might lead to an underestimation of the potential risk these agents pose to canine health and, by extension, to public health. Furthermore, the presence of inconclusive results underscores the need for more sensitive and specific diagnostic methods. This is crucial for implementing more effective prevention and control strategies, especially in shelter environments where the risk of disease transmission is heightened. Although inconclusive results do not significantly alter the observed trends in the study, they emphasise the importance of careful data interpretation and the need for continued research to refine diagnostic techniques and improve the epidemiological understanding of these infections.

The sensitivity of detecting *Rickettsia* spp. in blood appears to be moderate to low in humans [66,67] and dogs [15,68]. The differences between serological and molecular tests are likely due to *Rickettsia* spp. circulating in low levels in the blood during the acute phase of illness [69] and being promptly removed from blood. Experimental infections of dogs with *R. conorii* resulted in a brief rickettsiaemia lasting 2–10 days [70,71], which did not return even after immunosuppression [72].

The prevalence of ehrlichiosis is most noteworthy in regions characterised by a high concentration of primary population vectors, specifically “hard” ticks belonging to the genera *Rhipicephalus, Amblyomma* or *Dermacentor* [4]. The Mediterranean area offers an ideal habitat for the proliferation of numerous tick species. *Rhipicephalus sanguineus s. l.* and *Ixodes ricinus* are prevalent across Portugal and have been observed feeding on diverse hosts, including humans [73]. The same occurs for *R. conorii*, for which vectors are the primary route of infection, and infected ticks are likely the most important risk factor for infections in dogs and people [15]. However, in our study, no animal had ticks during blood collection. Since no ticks were observed, the low exposure of dogs in the two shelters, mainly to *R. sanguineus s. l.* ticks, associated with adequate tick control programs, may explain the low seroprevalence in this study.

Because dogs in this study were apparently healthy, positive animals were most likely in the chronic phase of infection [74]. Shelters can serve as a public health warning system for zoonotic diseases like those caused by *E. canis* and *R. conorii*. Positive serological results in dogs serve as a valuable warning system for veterinarians. These results may indicate prior exposure to a pathogen. While they do not confirm the presence of disease, they trigger a need for further investigation to ensure the welfare of the individual dog. This proactive approach to healthcare helps veterinarians promptly address any underlying health issues, contributing to their canine patients’ overall health and welfare [26].

Despite the rigorous analysis and methodology applied, this study has some limitations due to its cross-sectional nature and the limited number of shelters sampled, which may affect the generalisation of the results. The wide CI of the generated OR and the lack of travel histories for the dogs (stray animals) also constrain our ability to definitively attribute exposure to infected ticks to the shelter’s location. The findings were considered preliminary until confirmed by molecular techniques, which can provide a high level of specificity in determining the particular rickettsial species responsible for these antibody responses. Moreover, the potential for self-selection bias in a shelter-based study should be considered, as the exposure levels to vectors in shelter dogs might differ from those in domestic dogs.

Dogs kept at animal shelters are well known for harbouring and transferring virulent pathogens to animals and humans. Because of their origin as unwanted animals, filthy living circumstances in shelters, high population density, stress and exposure to rodents and arthropod vectors, shelter dogs are excellent sentinels for several vector-borne and zoonotic diseases [26,75]. In our study, the seroprevalence values observed for *E. canis* were lower than those reported previously in owned dogs. This finding concerning lower seropositivity in shelters is quite surprising, as we could expect that those who live in shelters had high seropositivity. Due to increased environmental exposure and a lack of preventatives, dogs that do not receive veterinary care and those who enter shelters, of which strays make up most cases, are likely more susceptible to contracting CVBD [75,76,77].

## 5. Conclusions

The findings of this study show that shelter dogs in northern Portugal are exposed to *E. canis* and *R. conorii,* which can affect both canines and humans. A One Health approach is necessary in order to educate the public about the hazards of canine zoonoses and develop legislation and procedures to control their spread and preserve public health. The regular screening of shelter animals can provide valuable data on the distribution and prevalence of rickettsial diseases, serving as sentinels to public health. This information should be used for epidemiological studies and surveillance, helping to understand disease patterns and to improve prevention strategies.

## Figures and Tables

**Table 1:** Seropositivity to *Ehrlichia canis* and *Rickettsia conorii* infections in two shelter dog populations prepared to be adopted (*n* = 113 dogs) from northern Portugal.

Pathogen	No. of Infected Dogs	Inconclusive Results	Prevalence (%)	95% CI ^a^
*E. canis*	1	4	0.9	0.02–4.8
*R. conorii*	11	12	9.7	4.9–16.8
Co-infection	12	16	10.6	5.6–17.8

^a^ 95% confidence interval.

**Table 2:** Seroprevalence of infection by *Ehrlichia canis*, *Rickettsia conorii* and co-infection in shelter dogs from northern Portugal.

Variables/Categories	*E. canis*—Positive/ Total (%)	95% CI ^a^	*R. conorii*—Positive/Total (%)	95% CI	Co-Infection (*E. canis* + *R. conorii*)—Positive/Total (%)	95% CI
**Sex**	*p* = 0.173		*p* = 0.003 *		*p* = 0.001 *	
Male	0/68 (0.0%)	0.0–5.3%	2/68 (2.9%)	0.36–10.2%	2/68 (2.9%)	0.36–10.2%
Female	1/45 (2.2%)	0.06–11.8%	9/45 (20.0%)	9.6–34.6%	10/45 (22.2%)	11.2–37.1%
**Age**	*p* = 0.235		*p* = 0.106		*p* = 0.057	
≤12 months	0/57 (0.0%)	0.0–6.3%	3/57 (5.3%)	1.1–14.6%	3/57 (5.3%)	1.1–14.6%
>12 months	1/56 (1.8%)	0.04–9.6%	8/56 (14.3%)	6.4–26.2%	9/56 (16.1%)	7.6–28.3%
**Origin**	*p* = 0.312		*p* ≤ 0.000 *		*p* ≤ 0.000 *	
Shelter 1	1/68 (1.5%)	0.04–7.9%	0/68 (0.0%)	0.0–5.3%	1/68 (1.5%)	(0.04–7.9%)
Shelter 2	0/45 (0.0%)	0.0–7.9%	11/45 (24.4%)	12.9–39.5%	11/45 (24.4%)	12.9–39.5%

^a^ 95% confidence interval; * *p* < 0.05.

**Table 3:** Univariable model for *Rickettsia conorii* and the co-infection (*E. canis* + *R. conorii*) in sheltered dogs.

Dependent Variable/ /Risk Factor	*p*-Value	OR ^a^	95% CI ^b^
Positivity to *R. conorii*			
**Sex**	*p* = 0.003		
Male		1	
Female		2.32	1.58–3.39
**Origin**	*p* ≤ 0.000		
Shelter 1		1	
Shelter 2		3.0	2.28–3.95
Positivity to mixed infection			
**Sex**	*p* = 0.001		
Male		1	
Female		2.4	1.66–3.47
**Origin**	*p* ≤ 0.000		
Shelter 1		1	
Shelter 2		2.72	1.97–3.76

^a^ Odds ratio; ^b^ 95% confidence interval.

**Table 4:** Risk factors associated with *Rickettsia conorii* and the co-infection (*Ehrlichia canis* + *R. conorii*) in sheltered dogs in multiple logistic regression analysis.

Risk Factor	β ^a^	S.E. β ^b^	*p*-Value	Adjusted OR ^c^	95% CI ^d^ (OR)
**Risk factor for *R. conorii***					
**Sex**	1.861	0.856	0.030		
Male				1	
Female				6.429	1.201–34.407
**Risk factors for mixed infection**					
**Sex**	2.029	0.836	0.015		
Male				1	
Female				7.606	1.478–39.132
**Origin**	2.903	1.801	0.007		
Shelter 1				1	
Shelter 2				18.229	2.190–151.756

^a^ Beta coefficient; ^b^ Standard error for β; ^c^ Odds ratio; ^d^ 95% confidence interval.

## Data Availability

The data presented in this study are available upon request from the corresponding author.
